# Supplementary material for: Effect of pregnancy and hypertension on kidney function in female rats: Modeling and functional implications
Source: PLoS One. 2023 May 30;18(5):e0279785. doi: 10.1371/journal.pone.0279785 (PMC10228769; doi:10.1371/journal.pone.0279785)
Supplement: S1 Text — Detailed description of juxtamedullary vs. superficial nephrons and pregnancy-specific model adaptations. (DOCX) [file pone.0279785.s001.docx]

**Supplementary Materials:** Effect of pregnancy and hypertension on kidney function in female rats: Modeling and functional implications

Melissa M. Stadt^1^, Crystal A. West^2^, and Anita T. Layton^1,3^

^1^ Department of Applied Mathematics, University of Waterloo, Waterloo, Ontario, N2L 3G1, Canada

^2^ Department of Biology, Appalachian State University, North Carolina Research Campus, Kannapolis, NC, 28081, USA

^3^ Department of Biology, Cheriton School of Computer Science, and School of Pharmacology, University of Waterloo, Waterloo, Ontario, N2L 3G1, Canada

**Supplemental Materials and Methods**

***Juxtamedullary vs. superficial nephrons.*** The length of the long descending limbs and ascending limbs are determined by which type of juxtamedullary nephron is being modeled. To capture this, in the model there are six classes of nephrons: a superficial nephron (denoted by “SF”) and five juxtamedullary nephrons that are assumed to reach depths of 1, 2, 3, 4, and 5 mm (denoted by “JM-1”, “JM-2”, “JM-3”, “JM-4”, and “JM-5”, respectively) based on the length of the long descending limb. As derived in Ref. [1], the ratios for the six nephron classes are n_SF=2/3, n_(JM-1)=0.4/3, n_(JM-2)=0.3/3, n_(JM-3)=0.15/3, n_(JM-4)=0.1/3, and n_(JM-5)=0.05/3 so that 2/3 of the nephrons are superficial. Note that shorter juxtamedullary nephrons are the most common, thus turning in the upper portion of the inner medulla. All other segments in the juxtamedullary nephrons are the same as the superficial nephrons except the length of the cortical thick ascending limb and the connecting tubule. Since the glomeruli of juxtamedullary nephrons are located lower in the cortex than the superficial nephron glomeruli, these segments do not have to be as long for the nephron to pass the glomerulus at the macula densa. Hence, the cortical thick ascending limb and connecting tubule are modeled with a shorter length.

Additionally, it has been shown that the SNGFR for juxtamedullary nephrons is higher than the superficial nephron SNGFR [1,2]. We assume that the juxtamedullary SNGFR is about 40% higher than the superficial SNGFR as in the virgin model [3].

The connecting tubules of the five juxtamedullary nephron types and the superficial nephron coalesce into the cortical collecting duct. To model this, we compute the flows from the six nephrons at the start of the collecting duct. The remaining model is the collecting duct which does not have distinct nephron segments. See Ref. [1] for more details on multi-nephron model development.

***Pregnancy-specific models.*** We created pregnancy-specific models to simulate kidney function in mid-pregnancy (MP) and late pregnancy (LP) by using the virgin (female-specific) multiple nephron epithelial transport model developed in Ref. [3] and increasing or decreasing relevant virgin model parameter values based on experimental findings in the literature. For changes in transporter activities for the MP and LP models we follow the same approach as in our previous study (Ref. [4]). These changes are briefly described below and discussed in more details in Ref. [4]. Because transporter activity changes in pregnancy are driven primarily by hormonal adaptations, we assumed that the pregnancy-induced changes in transporter activity levels in the juxtamedullary nephrons are the same as the superficial nephrons.

In pregnancy, kidney volume increases [5,6]. In particular, the proximal tubule, the first segment along the nephron where most Na^+^ and K^+^ reabsorption occurs, lengthens. Thus, we increased the proximal tubule length in the MP and LP models based on existing experimental measurements from Ref. [6,7]. We also assume an increase in the diameter along the nephron, based on observed dilation in the collecting ducts during pregnancy [8]. Without assuming a small tubular dilation, the much-elevated volume flow induced by the increased filtration would cause an excessive drop in tubular fluid pressure. Together increased proximal tubule length and nephron diameter result in a larger kidney volume.

We used experimental data to determine changes in parameter values for Na^+^ transporters in our MP and LP models. Much of these results are reviewed in Ref. [9]. Mahaney et al. [10] demonstrated region-specific changes in Na^+^-K^+^-ATPase activity and expression during pregnancy: Na^+^-K^+^-ATPase activity decreased throughout pregnancy in the cortex, but in the medulla, activity increase in MP while remaining unchanged in LP compared to virgin control [10]. Recently, West et al. [11] reported that the activity of Na^+^-K^+^-2Cl^-^ cotransporter 2 (NKCC2), a key transporter along the thick ascending limb, was significantly increased in LP and only slightly in MP. In the distal segments, the epithelial Na^+^ channel (ENaC) fine tunes the remaining Na^+^ in the luminal fluid before being excreted into urine. West et al. [12] reported that ENaC activity is nearly doubled in both MP and LP. Later findings showed renal adaptations in the ENaC are essential for sufficient Na^+^ retention during pregnancy [13]. In another study, West et al. [14] reported that the activity of the Na^+^-Cl^-^ cotransporter (NCC), which is found in the distal segments, is largely unchanged during MP and decreased during LP. Expression of the Cl-/bicarbonate exchanger, pendrin, in the connecting tubule and cortical collecting duct, is increased through pregnancy [15].

In the proximal tubule, the Na^+^/H^+^ exchanger (NHE3) drives much of the Na^+^ reabsorption. However, Na^+^/H^+^ exchanger activity has not been well characterized during pregnancy. We note that it has been shown that in female rats (i.e., virgin), there is higher protein expression but lower activity of NHE3 when compared with male rats, indicating reserve NHE3 that can be activated during pregnancy [3,16,17]. To avoid excess natriuresis, kaliuresis, and diuresis during pregnancy, we increased NHE3 activity in the MP and LP models based on the assumption that the reserve NHE3 in female rats is activated during pregnancy (see Table 1). An analysis of this assumption is discussed further in the Results and Discussion segments.

Aquaporin 2 (AQP2), the water channel in the collecting duct, is upregulated during MP and LP [18–20]. It has also been shown that the water channel in the descending limb, AQP1, is upregulated during LP, but not significantly changed during MP [19]. Based on these findings, water permeability in relevant segments was modified for increased AQP1 and AQP1 (see Table 1).

Since K^+^ retention starts during LP, changes in K^+^-specific renal transporters have mainly been studied during LP [9,21]. The K^+^-secretory channels in the distal segments, namely, the renal outer medullary K^+^ channel (ROMK) and large-conductance K^+^ channel (BK), are significantly downregulated, while H^+^-K^+^-ATPase pump activity is substantially increased during LP [21]. We increased H^+^-K^+^-ATPase activity and decreased K^+^ apical permeability in the appropriate distal segments in the LP model accordingly (see Table 1). Additionally, we hypothesized that the K^+^-Cl^-^ cotransporter in the ascending limb and distal convoluted tubule is upregulated during pregnancy to avoid excessive kaliuresis and natriuresis. While H^+^-K^+^-ATPase activity or BK permeability during MP remains poorly characterized, we note that Abreu et al. [18] showed that mRNA expression of ROMK2 is massively downregulated during MP. To avoid excessive kaliuresis and natriuresis in the MP model, we assume that similar changes to the K^+^ transporters occur during MP.

**References**

1. Layton AT, Vallon V, Edwards A. A computational model for simulating solute transport and oxygen consumption along the nephrons. Am J Physiol Renal Physiol. 2016;311(6):F1378–90.

2. Jamison RL. Intrarenal heterogeneity: The case for two functionally dissimilar populations of nephrons in the mammalian kidney. Am J Med. 1973 Mar 1;54(3):281–9.

3. Hu R, McDonough AA, Layton AT. Sex differences in solute transport along the nephrons: effects of Na+ transport inhibition. Am J Physiol Renal Physiol. 2020;319(3):F487–505.

4. Stadt MM, Layton AT. Adaptive changes in single-nephron GFR, tubular morphology, and transport in a pregnant rat nephron: modeling and analysis. Am J Physiol-Ren Physiol. 2022 Feb;322(2):F121–37.

5. J M Davison, M D Lindheimer. Changes in renal haemodynamics and kidney weight during pregnancy in the unanaesthetized rat. J Physiol. 1980;301(1):129–36.

6. Garland HO, Green R, Moriarty RJ. Changes in Body Weight, Kidney Weight and Proximal Tubule Length during Pregnancy in the Rat. Kidney Blood Press Res. 1978;1(1):42–7.

7. Atherton JC, Pirie SC. The effect of pregnancy on glomerular filtration rate and salt and water reabsorption in the rat. J Physiol. 1981 Jan 23;319:153–64.

8. Odutayo A, Hladunewich M. Obstetric Nephrology: Renal Hemodynamic and Metabolic Physiology in Normal Pregnancy. Clin J Am Soc Nephrol. 2012 Dec;7(12):2073–80.

9. de Souza AMA, West CA. Adaptive remodeling of renal Na+ and K+ transport during pregnancy. Curr Opin Nephrol Hypertens. 2018 Sep;27(5):379–83.

10. J Mahaney, C Felton, D Taylor, W Fleming, J Q Kong, C Baylis. Renal cortical Na+-K+-ATPase activity and abundance is decreased in normal pregnant rats. Am J Physiol - Ren Physiol. 1998;275(5):812–7.

11. West CA, Beck SD, Masilamani SME. Time course of renal sodium transport in the pregnant rat. Curr Res Physiol. 2021 Jan 1;4:229–34.

12. West C, Zhang Z, Ecker G, Masilamani SME. Increased renal alpha-epithelial sodium channel (ENAC) protein and increased ENAC activity in normal pregnancy. Am J Physiol Regul Integr Comp Physiol. 2010;299(5):R1326–32.

13. West CA, Han W, Li N, Masilamani SME. Renal epithelial sodium channel is critical for blood pressure maintenance and sodium balance in the normal late pregnant rat: Renal epithelial sodium channel blockade in late pregnancy. Exp Physiol. 2014;99(5):816–23.

14. West CA, McDonough AA, Masilamani SME, Verlander JW, Baylis C. Renal NCC is unchanged in the midpregnant rat and decreased in the late pregnant rat despite avid renal Na+ retention. Am J Physiol Renal Physiol. 2015;309(1):F63–70.

15. West CA, Verlander JW, Wall SM, Baylis C. The chloride–bicarbonate exchanger pendrin is increased in the kidney of the pregnant rat. Exp Physiol. 2015;100(10):1177–86.

16. Veiras LC, Girardi ACC, Curry J, Pei L, Ralph DL, Tran A, et al. Sexual Dimorphic Pattern of Renal Transporters and Electrolyte Homeostasis. J Am Soc Nephrol. 2017;28(12):3504–17.

17. Hu R, McDonough AA, Layton AT. Functional implications of the sex differences in transporter abundance along the rat nephron: modeling and analysis. Am J Physiol Renal Physiol. 2019;317(6):F1462–74.

18. Abreu N, Tardin JCBM, Boim MA, Campos RR, Bergamaschi CT, Schor N. Hemodynamic Parameters During Normal and Hypertensive Pregnancy in Rats: Evaluation of Renal Salt and Water Transporters. Hypertens Pregnancy. 2008;27(1):49–63.

19. Joyner J, Neves LAA, Stovall K, Ferrario CM, Brosnihan KB. Angiotensin-(1-7) serves as an aquaretic by increasing water intake and diuresis in association with downregulation of aquaporin-1 during pregnancy in rats. Am J Physiol - Regul Integr Comp Physiol. 2008;294(3):1073–80.

20. Ohara M, Martin PY, Xu DL, St John J, Pattison TA, Kim JK, et al. Upregulation of aquaporin 2 water channel expression in pregnant rats. J Clin Invest. 1998;101(5):1076–83.

21. West CA, Welling PA, West J, Coleman RA, Cheng KY, Chen C, et al. Renal and colonic potassium transporters in the pregnant rat. Am J Physiol Renal Physiol. 2018;314(2):F251–9.
